# Geroprotective potential of microbiome modulators in the *Caenorhabditis elegans* model

**DOI:** 10.1007/s11357-023-00901-7

**Published:** 2023-08-10

**Authors:** Brandi C. Miller, Megha Mathai, Hariom Yadav, Shalini Jain

**Affiliations:** 1 USF Center for Microbiome Research, Microbiomes Institute, University of South Florida, 12901 Bruce B Downs Blvd, MDC 78, Tampa, FL 33612 USA; 2 Department of Neurosurgery and Brain Repair, University of South Florida, Tampa, FL USA

**Keywords:** Geroscience, Aging, Microbiome, Gut, *C. elegans*, Probiotics, Postbiotics

## Abstract

Aging is associated with cellular and physiological changes, which significantly reduce the quality of life and increase the risk for disease. Geroprotectors improve lifespan and slow the progression of detrimental aging-related changes such as immune system senescence, mitochondrial dysfunction, and dysregulated nutrient sensing and metabolism. Emerging evidence suggests that gut microbiota dysbiosis is a hallmark of aging-related diseases and microbiome modulators, such as probiotics (live bacteria) or postbiotics (non-viable bacteria/bacterial byproducts) may be promising geroprotectors. However, because they are strain-specific, the geroprotective effects of probiotics and postbiotics remain poorly understood and understudied. *Drosophila melanogaster*, *Caenorhabditis elegans*, and rodents are well-validated preclinical models for studying lifespan and the role of probiotics and/or postbiotics, but each have their limitations, including cost and their translation to human aging biology. *C. elegans* is an excellent model for large-scale screening to determine the geroprotective potential of drugs or probiotics/postbiotics due to its short lifecycle, easy maintenance, low cost, and homology to humans. The purpose of this article is to review the geroprotective effects of microbiome modulators and their future scope, using *C. elegans* as a model. The proposed geroprotective mechanisms of these probiotics and postbiotics include delaying immune system senescence, preventing or reducing mitochondrial dysfunction, and regulating food intake (dietary restriction) and metabolism. More studies are warranted to understand the geroprotective potential of probiotics and postbiotics, as well as other microbiome modulators, like prebiotics and fermented foods, and use them to develop effective therapeutics to extend lifespan and reduce the risk of debilitating aging-related diseases.

## Introduction

Aging is defined by cellular, molecular, and physiological changes over time and is the major risk factor for many chronic and debilitating diseases like cancer, diabetes, and neurodegenerative diseases, which significantly reduce quality of life (QoL) and increase mortality [1, 2]. As the population continues to rapidly age, there is a dire need for therapeutics to reduce debilitating aging-related changes and the risk of disease. Geroprotective agents are primarily chosen because of their ability to (1) extend lifespan; (2) slow aging progression by reducing changes in cellular, molecular, and physiological biomarkers of aging; and (3) improve QoL during aging [3, 4]. They can impact aging biology by modulating immune responses to pathogens, quenching reactive oxygen species (ROS) and other mitochondrial byproducts, which damage lipids, proteins, and DNA, regulating mitochondrial function and stress responses, and regulating food intake (through dietary restriction [DR]) and metabolism [4]. However, the concept of geroprotection is emerging and we still lack fundamental knowledge of the precise molecular and physiological changes occurring during aging. As a result, there also remains a need to identify safe and effective geroprotectors to slow aging progression.

Our gut microbiota composition changes as we age, and emerging evidence suggests that significant perturbations in its composition and metabolites (dysbiosis) are a hallmark of aging-related diseases [5–9]. Therefore, maintaining gut microbiome health as we age is linked to a longer life, reduced disease risk, and improved QoL. The gut microbiome can be easily and beneficially modulated by many factors, including a fiber-rich diet, probiotics, prebiotics, and fermented foods; these changes are functionally stable, making the gut microbiome a promising and highly modifiable target for reducing aging-related disease burden [10]. Probiotics (live bacteria) and postbiotics (beneficial metabolites of probiotics or heat-inactivated bacteria) are microbiome modulators that may improve gut health and/or reduce the risk of debilitating aging-related diseases, and, thus, have geroprotective potential [11, 12]. Probiotics confer benefits when administered in adequate amounts and their effects are strain-specific [11, 13]. They must be (1) non-pathogenic and safe, (2) able to survive in acidic environments like the gut, and (3) be resistant against bile salts and enzymes [14]. Although many probiotics are also anti-pathogenic (i.e., against enteropathogens like *Helicobacter pylori* and *Campylobacter jejuni*) and have strong resistance against common antibiotics, these qualities are not required to be considered a probiotic [15, 16]. Potential benefits of probiotics include significant restoration of good bacteria and production of beneficial metabolites (i.e., short-chain fatty acids [SCFAs] like acetate and butyrate) [15, 17–19]. Multi-strain probiotics, like VSL#3 and fermented milk, improved obesity and diabetes via gut microbiome modulation and the production of SCFAs (i.e., butyrate) in animals and humans [20–26]. We have recently shown that a human-origin probiotic cocktail containing five lactobacilli and five enterococci improved aging-related leaky gut and inflammation in obese mice [27]. Other studies reported the beneficial effects of multi-strain probiotics on behavior, inflammation, oxidative stress, and gut microbiome composition in aging mice [28–30] and on the microbiome and cognitive functions in humans [31]. Compared to probiotics, the geroprotective potential of postbiotics is significantly less studied. We recently showed that heat-inactivated *Lactobacillus paracasei* D3.5 significantly ameliorated aging-related leaky gut and inflammation and restored physical and cognitive deficits in *Caenorhabditis elegans* and mice [32]. However, the anti-aging and geroprotective potentials of probiotics and postbiotics remain elusive and require further investigation.

There are multiple preclinical models available to study the effects of microbiome modulators on lifespan and geroprotection, including *Drosophila melanogaster* (fruit flies), *C. elegans* (nematodes), and rodents (mice/rats). The geroprotective effects of probiotics have been demonstrated in each of these models, but each model also has limitations, including their ability to recapitulate human aging biology and feasibility for use in larger-scale screening studies [33]. In this manuscript, we are focusing on the geroprotective effects of probiotics and postbiotics in *C. elegans,* which is a powerful model for large-scale and high-throughput screening studies due to its quick reproducibility and short lifespan (~ 2 weeks), low cost, easy maintenance, and close homology to humans [33].

**Fig. 1:**
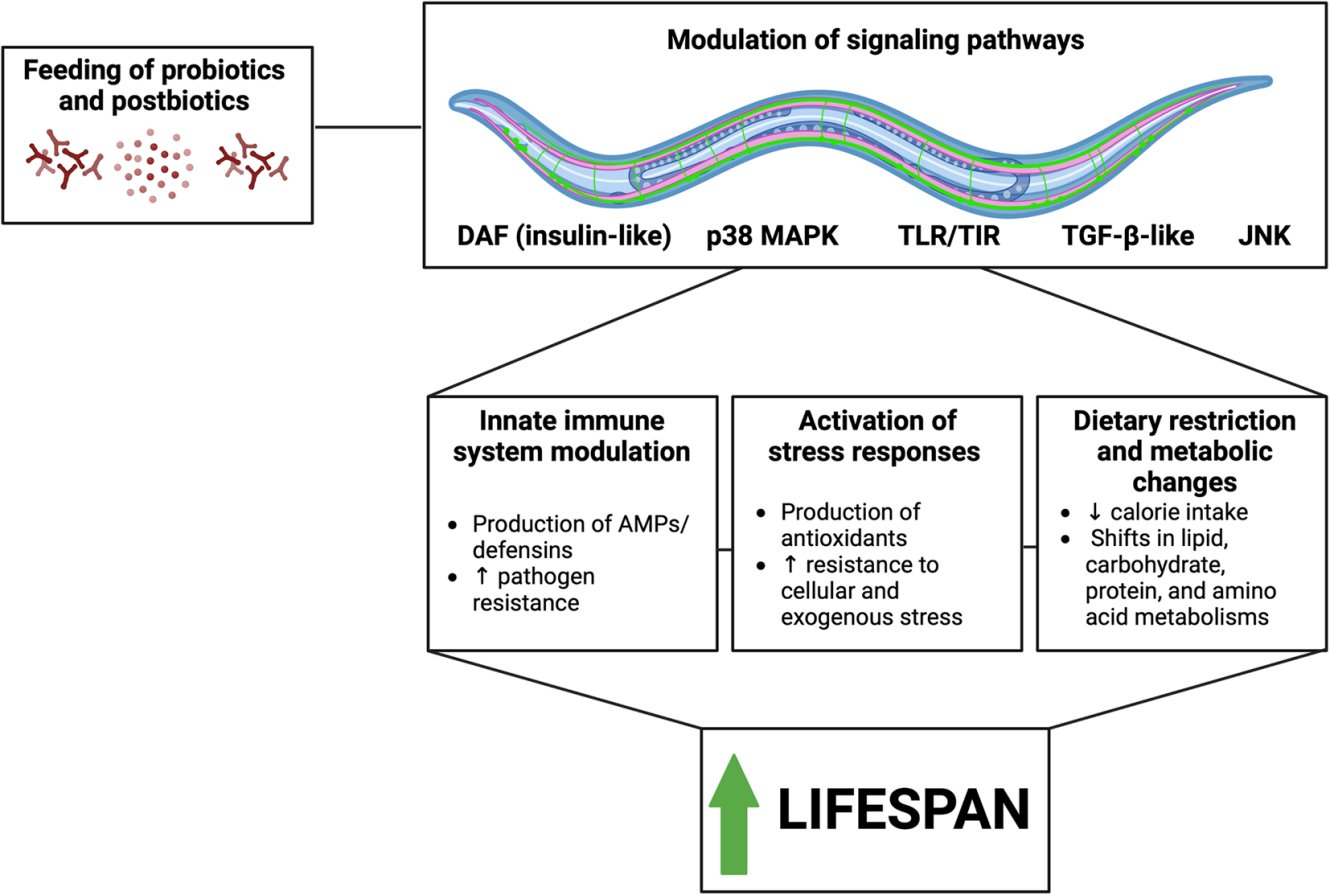
Supplementing probiotics and postbiotics to *C. elegans* significantly improves lifespan by targeting multiple pathways. These include the DAF (insulin-like), p38 MAPK, TLR/TIR, TGF-β-like, and JNK pathways, which modulate the innate immune system, activate stress responses, and change feeding patterns and host metabolism. AMPs, antimicrobial peptides; *C. elegans*, *Caenorhabditis elegans*; DAF, dauer formation; JNK, c-Jun N-terminal kinase; MAPK, mitogen-activated protein kinase; TGF-β, transforming growth factor-β; TIR, Toll-interleukin repeat; TLR, Toll-like receptor

According to metagenomic studies, the dauer formation (DAF), p38 mitogen-activated protein kinase (MAPK), and c-Jun N-terminal kinase (JNK) pathways are central to aging pathology in *C. elegans* [33, 34]. Details of these pathways have been described elsewhere [33, 35–37] and are largely outside of the scope of this review. Briefly, the DAF cascade, including the *daf-2* insulin-like receptor, *daf-16* master transcription factor, and several other *daf* genes, is activated in response to nutrient levels, which are connected to metabolism, growth, development, and behavior, and therefore impacts lifespan [35, 37]. In addition, the p38 MAPK pathway affects life- and healthspan in *C. elegans* via innate immune system modulation and by conferring resistance to pathogenic bacteria [36, 38]. The JNK pathway is activated in response to several stressors, such as oxidative stress, heat, ultraviolet irradiation, and inflammation [37]. Therefore, targeting these pathways using high-throughput screening pipelines may significantly contribute to our understanding of geroprotection and lifespan extension mediated by microbiome modulators [39]. According to studies investigating host–microbiome interactions in *C. elegans*, many probiotics colonize significantly in the worm gut and their signals may modulate the DAF, p38 MAPK, JNK, or other pathways by modulating innate immunity (by regulating signaling pathways or producing antimicrobial compounds), decreasing cellular stress (by reducing ROS through antioxidants or enhancing stress resistance), or regulating food intake through DR and shifting levels of beneficial metabolites [40, 41] (Fig. 1). The simplest approach for studying the geroprotective potential of microbiome modulators is to study lifespan, but other parameters like (1) body length, (2) body fat percentage, (3) pharyngeal pumping rate, (4) locomotion, (5) intestinal permeability, (6) brood size, and (7) muscle mass are also important for evaluating healthspan [42, 43]. Because the standard and most common laboratory diet for *C. elegans* is *Escherichia coli* OP50, with other common strains including *E. coli* K-12, *E. coli* HT115, and *E. coli* HB101 [44], screening the effects of potential probiotics only requires changing the diet [33]. Most studies have focused on single strains, such as those of genera *Lactobacillus*, *Bifidobacterium*, *Enterococcus*, and *Streptococcus*. The beneficial metabolites and byproducts of probiotics and heat-inactivated probiotics (both collectively referred to as postbiotics) are also being investigated to determine if bacteria in their non-viable form have geroprotective potential.

The purpose of this manuscript is to review the studies reporting the anti-aging effects and geroprotective potential of probiotics and postbiotics, focusing on their effects and proposed mechanisms in the *C. elegans* model.

## Advantages of using *C. elegans* for large-scale screening studies

*D. melanogaster*, *C. elegans*, and rodents are well-established and relevant models for studying host–microbiome interactions and evaluating the potential benefits of probiotics and postbiotics. Single- and/or multi-strain combinations of *Limosilactobacillus reuteri*,* Lactobacillus fermentum*, *Lactobacillus plantarum*, *Bifidobacterium longum* subsp. *infantis*, and *Enterococcus faecium* significantly extended lifespan in *D. melanogaster* via modulation of insulin-like genes (i.e., *dilp-2*) and reduction of ROS levels [45–47]. Furthermore, multiple bifidobacteria, including *Bifidobacterium breve*, *Bifidobacterium animalis*, *B. longum*, and *Bifidobacterium adolescentis* improved lifespan by reducing ROS accumulation or increasing expression of antioxidants as catalase (CAT, gene name *ctl*) and superoxide dismutase (SOD, gene name *sod*) [48, 49]. Despite these reports, studying the microbiome in *D. melanogaster* is limited because they have a low microbial diversity and high-throughput screening protocols are more challenging to implement [33]. Thus, *C. elegans* has emerged as powerful model organism for investigating host–microbiome interactions in larger-scale studies.

*C*. *elegans* are also widely used because of their low cost, quick development to reproductive adulthood (2–3 days), relatively short lifespan (2–3 weeks), and easy laboratory maintenance [33]. According to 16S rDNA sequencing, the *C. elegans* core microbiome changes depending on its natural environment and diet, is considerably species rich, and is characterized by many distinct taxa, with Proteobacteria and *Enterobacteriaceae* being the most abundant [50–53]. Under laboratory conditions, where *C. elegans* are most commonly fed *E. coli* OP50, their microbiome is rich in Proteobacteria, Firmicutes, and Actinobacteria (at the phylum level)—thus, when their diet is controlled in the laboratory setting, it allows us to easily understand how different microbes impact their life- and healthspan [54]. These reports highlight the strengths of using *C. elegans* to study host–microbiome interactions and also can recapitulate how introducing specific microbes can impact host processes in mammalian systems and how modulating these pathways may have geroprotective effects. Transcriptomic, proteomic, metabolomic, and functional analyses are available to use with *C. elegans*, allowing us to understand the molecular changes associated with lifespan extension and anti-aging physiology. Furthermore, *C. elegans* studies are the most feasible for large-scale screening studies, especially in the context of aging, compared to rodents. In the laboratory, mice and rats live 2–3 years and are costly, making screening studies in these organisms impractical and expensive [33, 55]. Based on these advantages in *C. elegans* and the limitations of other organisms, we are focusing on the geroprotective potential of probiotics and postbiotics using *C. elegans* as a model organism. However, it is important to validate the findings from *C. elegans* screening experiments in complex mammalian systems to better translate the effects of probiotics and/or postbiotics on the microbiome and disease to humans.

## Geroprotective effects of probiotics in *C. elegans*

*Lactobacillus* and *Bifidobacterium* have been studied the most as potential geroprotectors, but emerging screening studies are demonstrating that other genera, including *Enterococcus*, *Streptococcus*, and *Bacillus*, also have geroprotective effects in *C. elegans*. In these studies, geroprotection was assessed based on extended lifespan, in comparison to *E. coli* OP50 (hereafter referred to as OP50) or *E. coli* HB101, two common laboratory diets for *C. elegans*, or *L. rhamnosus* GG (LGG) as a positive control (due to its well-studied probiotic potential and ability to colonize in the *C. elegans* gut). Other anti-aging markers such as increased locomotion, decreased body fat/size and pharyngeal pumping, and reduced accumulation of ROS, lipofuscin (a product of lipid peroxidation), or lipid droplets were also observed in several studies. Probiotics impact lifespan and worm physiology/behaviors by modulating (1) the innate immune system, (2) stress responses, and (3) food intake and metabolism; many studies report modulation of multiple or all these systems, demonstrating potential multifactorial effects of probiotics. Table 1 summarizes the effects of *Lactobacillus* and *Bifidobacterium* strains on *C. elegans* lifespan*,* while Table 2 summarizes the effects of other genera on the lifespan in *C. elegans*.

**Table 1:** Beneficial effects of *Lactobacillus* and *Bifidobacterium* species on *Caenorhabditis elegans* lifespan and their proposed mechanism and pathway

Year	Species name(s)	Effects on *C. elegans* lifespan	Proposed mechanisms	Reference
*Lactobacillus*				
2007	*L. helveticus* *L. plantarum* *L. rhamnosus*	**• ↑** by 25%, 22%, and 33%, respectively **• ↑** by 46%, 35%, and 35% under *S. enterica* infection	*Mechanism:* immune system modulation *Pathway:* p38 MAPK, JNK-DAF	[57]
2012	*L. rhamnosus* CNCM I-3690	**• ↑** by 20% **• ↑** by 30% after H_2_O_2_-induced oxidative stress	*Mechanism:* stress response *Pathways:* DAF, SKN-1, JNK	[90]
2012	*L. acidophilus* NCFM	**• ↑** after infection with *E. faecalis* and *S. aureus*, including antibiotic-resistant strains	*Mechanism:* immune system modulation *Pathways:* p38 MAPK (*pmk-1*, *tir-1*) and BAR-1	[60]
2013	*L. salivarius* FDB89	**• ↑** by ≤ 11.9%	*Mechanisms:* stress response and DR	[115]
2014	*L. zeae* LB1	**• ↑** by 78% after infection with ETEC strain JG280	*Mechanism:* immune system modulation and inhibition of enterotoxin production	[80]
2014	Four *L. plantarum* (JDFM440, JDFM60*,*JDFM970*,* and JDFM1000)	**• ↑** lifespan **• ↑** resistance to *S. aureus* RN6390	*Mechanism:* immune system modulation	[40]
2016	*L. gasseri* SBT2055	**• ↑** lifespan (dose-dependently)	*Mechanism:* stress response *Pathways:* DAF and SKN-1	[92]
2016	*L. rhamnosus* R4 *L. helveticus* S4	**• ↑** (compared to OP50 and *E. hirae* H4) **• ↑** by 36.1% after *L. rhamnosus* R4 feeding	*Mechanisms:* stress response; improved ROS scavenging and cholesterol/triglyceride levels (in vitro)	[85]
2017	*L. delbrueckii* subsp. *bulgaricus*	**• ↑** (compared to two *L. delbrueckii* isolates and OP50)	*Mechanisms:* changes in metabolism (folate, amino acids, galactose, fatty acids)	[108]
2018	*L. zeae* LB1	**• ↑** (compared to *L. casei*)	*Mechanism:* Immune system modulation *Pathways:* p38 MAPK and DAF (insulin-like)	[65]
2018	*L. fermentum* JDFM216	**• ↑** lifespan **• ↑** resistance to food-borne pathogens *S. aureus* and *E. coli* O157:H7	*Mechanism:* immune system modulation *Pathways:* p38 MAPK (*pmk-1*) and nuclear hormone receptors	[63]
2019	*L. fermentum* MBC2	**• ↑** (compared to LGG and OP50)	*Mechanism*: stress response and metabolism *Pathway: pept-1* (lipid storage)	[76]
2020	*L. fermentum* U-21	**• ↑** by 27% **• ↑** after paraquat-induced oxidative stress	*Mechanism*: stress response	[91]
2020	*L. plantarum* 427 *L. crispatus* X13 *L. reuteri* 9–5 *L. fermentum* 422 *L. salivarius* Z5 *L. reuteri* G14	**• ↑** (ranging from 70 to 85%) **• ↑** after H_2_O_2_- and juglone-induced oxidative stress	*Mechanism:* stress response and increased antioxidant capacity *Pathways:* p38 MAPK and SKN-1/Nrf-2	[93]
2021	*L. sakei* 20D49	**• ↑** lifespan **• ↑** after *S. aureus* infection	*Mechanism:* immune system modulation	[75]
2021	*L. salivarius* 13–7 *L. salivarius* Z5 *L. plantarum* N9	**• ↑** by ≥ 40% after *C. jejuni* infection	*Mechanism:* immune system modulation *Pathways:* p38 MAPK and DAF	[61]
2021	*Lacticaseibacillus rhamnosus* GG	**• ↑** lifespan **• ↑** after *S. aureus*, *S. typhimurium*, and *E. faecalis* infections	*Mechanism*: immune system modulation *Pathways:* Wnt, TGF-β and MAPK (mediated by miRNA-34)	[77]
2021	*L. zeae*	**• ↑** after *S. typhimurium* DT104 infection	*Mechanism:* immune system modulation *Pathway:* p38 MAPK	[66]
2021 and 2022	*Lactobacillus* spp. Lb21 (mix of *Levilactobacillus brevis* and *Lactiplantibacillus plantarum*)	**• ↑ (**compared to other lactobacilli in a 93-strain screen) **• ↑** after MRSA infection	*Mechanism:* Immune system modulation and changes in metabolism (amino acids) *Pathways*: TGF-β	[69, 107]
2022	*L. plantarum* As21	**• ↑** by 34.5% **• ↑** after H_2_O_2_- and heat-induced cellular stress	*Mechanism:* stress response (reduced ROS and increased antioxidants)	[86]
2022	*L. plantarum* JBC5	**• ↑** by 27.81%	*Mechanisms:* immune system modulation, stress response, and changes in metabolism (lipids) *Pathway:* p38 MAPK (*sek-1*, *nsy-1*, *pmk-1*, *skn-1*)	[62]
2022	*Lacticaseibacillus rhamnosus* Probio-M9	**• ↑** by ~ 30%	*Mechanisms:* immune system modulation and stress response *Pathways:* p38 MAPK (*nsy-1*, *sek-1*, and *pmk-1*) and DAF	[78]
2022	*Lactiplantibacillus plantarum* P1	**• ↑** by 39.7% after *G. vaginalis* infection	*Mechanism:* immune system modulation	[54]
2023	*Levilactobacillus brevis*	**• ↑** by 15.9% **• ↑** by 45.1% and 69.2% following oxidative and heat stresses, respectively	*Mechanisms:* stress response and improved gut barrier functions/permeability *Pathways*: p38 MAPK, DAF (insulin-like), and JNK	[96]
*Bifidobacterium*				
2007	*B. infantis* *B. longum*	**• ↑** by 29% and 17%, respectively **• ↑** by 30% and 35% after *S. enterica* serovar Enteriditis infection	*Mechanism:* immune system modulation *Pathway:* p38 MAPK, JNK-DAF	[57]
2013	*B. infantis*	**• ↑** (dose-dependent)	*Mechanism:* cell wall components *Pathways:* p38 MAPK and DAF	[58]
2016	*B. animalis* subsp. *lactis* CECT 8145	**• ↑** by ~ 30%	*Mechanisms:* stress response and changes in metabolism (amino acids, fatty acids/lipids, carbohydrates, xenobiotics) *Pathway:* DAF (insulin-like)	[113]
2017	*B. longum* BB68	**• ↑** by 28%	*Mechanisms:* immune system modulation and stress response *Pathways:* TIR-1-JNK-1-DAF	[116]
2019	*B. infantis*	**• ↑** (compared to *B. subtilis* and *C. butyricum*)	*Mechanism:* immune system modulation *Pathway:* TOL-1 (TLR)	[72]
2021	*B. adolescentis*	**• ↑** lifespan **• ↑** after heat-induced cellular stress	*Mechanism:* stress response	[48]

BAR-1, β-catenin pathway; *B.*, *Bifidobacterium*; *B. subtilis*, *Bacillus subtilis*; *C. butyricum*, *Clostridium butyricum*; *C. jejuni*, *Campylobacter jejuni*; DAF, dauer formation; DR, dietary restriction; *E. coli*, *Escherichia coli*; *E. faecalis*, *Enterococcus faecalis*; *E. hirae, Enterococcus hirae*; ETEC, enterotoxigenic *Escherichia coli*; *G. vaginalis*, *Gardnerella vaginalis*; H_2_O_2_, hydrogen peroxide; JNK, c-Jun N-terminal kinase; *L.*, *Lactobacillus*; LGG, *Lactobacillus rhamnosus* GG; MAPK, mitogen-activated protein kinase; miRNA, micro-RNA; MRSA, methicillin-resistant *Staphylococcus aureus*; Nrf-2, nuclear factor erythroid 2-related factor 2; OP50, *Escherichia coli* OP50; ROS, reactive oxygen species; *S. aureus, Staphylococcus aureus*; *S. enterica*, *Salmonella enterica*; SKN-1, Skinhead-1; *S. typhimurium*, *Salmonella typhimurium*; TGF-β, transforming growth factor-β; TIR-1, Toll/interleukin-1 receptor; TLR, Toll-like receptor

**Table 2:** Beneficial effects of other genera on *Caenorhabditis elegans* lifespan and proposed mechanism and pathway

Year	Species name(s)	Effects on *C. elegans* lifespan	Proposed mechanism/pathway	Reference
*Enterococcus*				
2018	*E. faecium* L11	**• ↑** (compared to OP50) **• ↑** after *S. typhimurium* infection	*Mechanism:* immune system modulation *Pathways*: p38 MAPK, TGF-β, and DAF (insulin-like)	[73]
2021	*E. faecalis* 12D26 *E. faecalis* 20D48 *E. faecalis* 30D36	**• ↑** (compared to OP50) **• ↑** resistance to *S. aureus* infection **• ↑** resistance to *E. coli* O157:H7 (20D48 and 30D36 only)	*Mechanism*: immune system modulation	[75]
2022	*E. faecium*	**• ↑** resistance to *S. enterica* infection	*Mechanism:* immune system modulation, mediated by HLH-26 (transcription factor) *Pathway:* Wnt/BAR-1	[83]
*Weisella*				
2015	*W. koreensis* *W. cibaria*	**• ↑** (compared to OP50)	*Mechanism:* stress response *Pathways:* DAF and JNK	[94]
2022	*W. confusa*	**• ↑** (compared to OP50) **• ↑** resistance to H_2_O_2_-induced oxidative stress and *S. typhimurium* infection	*Mechanisms:* immune system modulation and stress response, observed through RNAseq/gene expression analyses	[117]
*Bacillus*				
2015	*B. licheniformis* 141 *B. licheniformis* 143 *B. licheniformis* 147 *B. licheniformis* 156	**• ↑** (compared to OP50 and *B. subtilis* ATCC 6633 controls)	*Mechanism:* serotonin signaling, observed through gene expression analyses	[120]
2017	*B. subtilis* JH642 *B. subtilis* NCIB3610 *B. subtilis* RG4365	**• ↑** by 23.5% (JH642), 52.3% (NCIB3610), and 59.5% (RG4365)	*Mechanism:* biofilm formation and NO production	[122]
2020	*B. subtilis* PXN21 *B. subtilis* JH642 *B. subtilis* 168 *B. subtilis* NCIB3610	**• ↑** in α-synuclein worms	*Mechanism:* DR, biofilm formation, and NO production *Pathways:* DAF-16 (vegetative cells), PHA-4/DR (spores)	[105]
2020	*B. subtilis*	**• ↑** in transgenic worms overexpressing Aβ	*Mechanism:* biofilm formation	[123]
*Propionibacterium*				
2016	*P. freudenreichii*	**• ↑** by ~ 13% (compared to controls) **• ↑** resistance to *S. typhimurium*	*Mechanisms*: immune system modulation, and upregulation of antimicrobial genes *Pathways:* p38 MAPK, TGF-β, DAF (insulin-like)	[74]
*Pediococcus*				
2018	*P. acidilactici* DM-9 *L. brevis* SDL1411 *P. pentosaceus* SDL1409	**• ↑** lifespan **• ↑** resistance against *P. aeruginosa* PA14 infection	*Mechanisms:* immune system modulation, NO production	[81]
2022	*P. acidilactici*	**• ↑** lifespan	*Mechanisms:* immune system modulation, stress response, changes in metabolism (lipids) *Pathways:* p38 MAPK, JNK, DAF (insulin-like)	[119]
2022	*P. acidilactici* CECT9879	**• ↑** lifespan	*Mechanisms:* stress response and changes in metabolism (fatty acid degradation and synthesis) *Pathway:* DAF (insulin-like)	[114]
2022	*P. acidilactici* MNL5	**• ↑** lifespan (also in *daf-2* and *liu1*) transgenic worms	*Mechanisms:* DR and changes in metabolism (lipids)	[104]
*Lactococcus*				
2022	*L. cremoris* subsp. *cremoris*	**• ↑** lifespan **• ↑** resistance against *S. enteriditis* and* S. aureus*	*Mechanisms:* immune system modulation, stress response, decreased gut permeability *Pathways:* p38 MAPK (*skn-1* and *pmk-1*) and DAF	[118]
*Streptococcus*				
2021	*S. thermophilus* ST-T1 *S. thermophilus* ST-510	**• ↑** lifespan (compared to OP50)	*Mechanisms:* stress response and increased antioxidants, observed through changes in expression of related genes *Pathway:* DAF	[95]
*Butyricioccus*				
2018	*B. pullicaecorum*	**• ↑** by 6.9% **• ↑** resistance to *S. typhimurium*	*Mechanism:* immune system modulation *Pathway:* TGF-β (*dbl-1*)	[70]
*Megasphaera*				
2018	*M. elsdenii*	**• ↑** by 6.9% **• ↑** resistance to *S. typhimurium*	*Mechanism:* immune system modulation *Pathway:* TGF-β (*dbl-1*)	[70]
*Clostridium*				
2018	*C. butyricum* MIYAIRI 588	**• ↑** (MIYAIRI 588 alone and in combination with OP50) **• ↑** resistance to *S. enterica* or *S. aureus* infections and UV irradiation–induced cellular stress	*Mechanisms:* immune system modulation, stress resistance, DR, and changes in metabolism *Pathway:* DAF	[67]
*Leuconostoc*				
2021	*L. mesenteroides* C2 *L. mesenteroides* C7	**• ↑** by ~ 50% **• ↑** resistance to *P. aeruginosa* and *S. aureus*	*Mechanisms:* immune system modulation *Pathway:* p38 MAPK (*pmk-1* and *hsf-1*)	[64]
*Stenotrophomonas*				
2021	*Stenotrophomonas* strain CPCC 101271	**• ↑** by 40% (compared to OP50) **• ↑** resistance to *B. nematocida* B16	*Mechanism:* immune system modulation	[79]
*Weizmannia*				
2023	*W. coagulans*	**• ↑** by 34.3% **• ↑** by 67.7% and 78.8% following oxidative and heat stresses, respectively	*Mechanisms:* stress response and improved gut barrier functions/permeability *Pathways*: p38 MAPK, DAF (insulin-like), and JNK	[96]

Aβ, amyloid beta; BAR-1, β-catenin pathway; *B. nematocida, Bacillus nematocida*; DAF, dauer formation; DR, dietary restriction; *E. coli*, *Escherichia coli*; HLH-26, helix-loop-helix 26 transcription factor; H_2_O_2_, hydrogen peroxide; JNK, c-Jun N-terminal kinase; MAPK, mitogen-activated protein kinase; NO, nitric oxide; OP50, *Escherichia coli* OP50; *P. aeruginosa*, *Pseudomonas aeruginosa*; PHA-4, defective pharynx development; *S. aureus, Staphylococcus aureus*; *S. enterica*, *Salmonella enterica*; *S. enteriditis*, *Salmonella enteriditis*; *S. typhimurium*, *Salmonella typhimurium*; SKN-1, Skinhead-1; SOD, superoxide dismutase; TGF-β, transforming growth factor-β

### Modulation of innate immune system

Immune system impairment occurs during aging, leaving us more susceptible to infections, which can severely impact QoL and even be lethal. Therefore, modulating the immune system is one mechanism by which probiotics may improve lifespan and slow aging progression. Under *Gardnerella vaginalis* infection, *Lactiplantibacillus plantarum* P1 improved the *C. elegans* lifespan by 39.7% compared to OP50-fed controls [56]. Although the mechanism was not explored in vivo, *L. plantarum* P1 had several characteristics of common probiotics, including tolerance to low pH and bactericidal activity, and, because *G. vaginalis* is a potent pathogen and the causative agent of bacterial vaginosis, it is inferred that immune system modulation had a prominent role in *L. plantarum* P1-mediated geroprotection [56]. While *C. elegans* lack many fundamental immune system mediators like nuclear factor kappa B (NFκB) and myeloid differentiation primary response 88 (MyD88), other evolutionarily conserved pathways including p38 MAPK, transforming growth factor β (TGF-β)-like, insulin-like, and toll-like receptor (TLR) pathways are central to their innate immune system [57, 58]. An early study reported that feeding with *Lactobacillus helveticus*, *L. plantarum*, *Lactobacillus rhamnosus*, *B. infantis*, and *B. longum* increased worm lifespan by 25%, 22%, 33%, 29%, and 17%, respectively, compared to control worms and significantly improved resistance to *Salmonella enterica* serovar *Enteritidis* infection [59]. The mechanisms contributing to improved lifespan were not investigated, but the authors speculated that bacterial cell wall components may have modulated DAF or p38 MAPK signaling and stimulated the immune system, thus enhancing pathogen resistance. These authors later reported that *B. infantis* whole cell and cell wall components improved lifespan through *pmk-1* (MAPK) and *skn-1* (skinhead-1), two critical genes in the p38 MAPK cascade [60]. *skn-1* is an ortholog of human NF-E2-related factor (*nrf-2*) and is involved in many cellular processes, such as stress response, detoxification, lipid metabolism, and immunity [61]. Other studies have also confirmed the importance of the p38 MAPK signaling pathway and its regulators, including *pmk-1*, *sek-1* (encodes MAPKK), *nsy-1* (encodes MAPKKK), and *tir-1* (toll-interleukin repeat protein; contains a toll-like receptor domain), in lifespan extension and pathogen resistance. Supplementation of *Lactobacillus acidophilus* NCFM improved resistance to *Enterococcus faecalis* and *Staphylococcus aureus* infections at least in part via *pmk-1* and *tir-1* [62]. NCFM also modulated the β-catenin (BAR-1) pathway, which is highly conserved in *C. elegans* and integral to Wnt signaling [62]. *Lactobacillus salivarius* (13–7 and Z5), *L. plantarum* N9, and *L. plantarum* JBC5 feeding significantly upregulated the expression of p38 MAPK genes, specifically *nsy-1*, *sek-1*, and *pmk-1* [63, 64]. Deletion of *pmk-1* significantly reduced *C. elegans* lifespan, even with *L. fermentum* JDFM216 pre-treatment, suggesting that *pmk-1* (and nuclear hormone receptors, which are affected downstream of *pmk-1* phosphorylation) are central mediators of longevity under exposure to food-borne pathogens like *S. aureus* and *E. coli* O157:H7 [65]. Newly identified *Leuconostoc mesenteroides* (strains C2 and C7) significantly improved lifespan and resistance to *S. aureus* and *Pseudomonas aeruginosa*; upregulated *pmk-1* and *hsf-1* (activated in response to heat shock) transcripts suggested that *L. mesenteroides* strains enhanced longevity through p38 MAPK signaling [66].

Furthermore, the insulin-like pathway, which includes the insulin-like receptor (*daf-2*) and its transcriptional regulator (*daf-16*) has impacts on lifespan in the context of pathogen exposure and *C. elegans* innate immunity [35]. *Lactobacillus zeae* LB1 impacted *daf* signaling, as mutants defective in *daf-16* were significantly more susceptible to enterotoxigenic *E. coli* (ETEC) infection [67, 68]. *skn-1* mutants were more susceptible to *S. aureus* infection, even after feeding with *L. plantarum* JBC5, indicating at least a partial dependence of insulin-like signaling on lifespan extension during infection [64]. Real-time PCR also revealed that *daf-16*, as well as other genes in the DAF pathway (*skn-1, age-1*, or *daf-2*) were significantly upregulated after pre-treatment with *L. salivarius* (13–7 or Z5) or *L. plantarum* N9 (under *C. jejuni* infection) [63] or *Clostridium butyricum* MIYAIRI 588 (under *S. enterica* or *S. aureus* infections) [69].

DBL-1 is a ligand in the TGF-β-like pathway, which is anti-inflammatory and contributes to innate immunity/antimicrobial activity in *C. elegans*, and there are several *sma* genes (human *smad* orthologs) activated downstream of DBL-1 [70]. Feeding of *Lactobacillus* spp. Lb21 (containing a mix of *Levilactobacillus brevis* and *Lactiplantibacillus plantarum*) improved resistance to methicillin-resistant *S. aureus* (MRSA), including clinical isolates, through a *dbl-1* mediated mechanism in the intestine [71]. Mutant worms defective in *dbl-1* or *daf-12* had a shortened lifespan under *Salmonella typhimurium* infection, even after treatment with *Butyricicoccus pullicaecorum* or *Megasphaera elsdenii*, indicating a central role of *dbl-1* signaling and TGF-β [72].

In *C. elegans*, the sole TLR is encoded by *tol-1* and worms defective in this gene are highly susceptible to Gram-negative pathobionts like *S. enterica* but are more resistant to Gram-positive microbes like *E. faecalis* [73]. Nonetheless, its role in probiotic-mediated longevity and geroprotection require further investigation. Interestingly, *tol-1* mutant worms were significantly more resistant to *E. faecalis* and *S. aureus* (both Gram-positive) infections, which was enhanced by *B. infantis* feeding [74]. Innate immunity-related genes, such as those encoding lysozyme/invertebrate-type lysozymes (*lys-3*, *ilys-2*, and *ilys-3*) were significantly upregulated in *tol-1* mutants, suggesting that *tol-1* could be central to immune responses to Gram-negative bacteria, rather than Gram-positive bacteria [74]. The authors also hypothesized that expression of *tol-1* promotes the digestion of the *B. infantis* cell wall (as it is Gram positive); nonetheless, the mechanisms remain largely unexplored and require further investigation.

As these three pathways are tightly regulated and are central to the *C. elegans* innate immune system, they are likely all interconnected and contribute to anti-pathogenicity and lifespan protection elicited by probiotics. *L. zeae* LB1 and *E. faecium* L11 both modulated MAPK pathways (i.e., *pmk-1* or *sek-1*) and the insulin-like pathway (i.e., *daf-2* or *daf-16*) under *S. typhimurium* infection; this was determined directly using mutant worms defective in these genes or by observing a positive correlation between gene expression and lifespan extension/pathogen resistance [67, 68, 75]. *E. faecium* L11 also increased gene expression of *dbl-1* and *sma-3*, suggesting modulation of the TGF-β-like pathway and downstream human SMAD orthologs, and was shown to activate macrophages in vitro, further highlighting the importance of this probiotic in innate immune system activation [75]. Resistance to *S. typhimurium* by *Propionibacterium freudenreichii* was shown to modulate the p38 MAPK, TGF-β-like, and insulin-like pathways by using mutant worms for lifespan assays and observing increases in gene expressions of *daf*, *pmk-1*, and *sek-1* (p38 MAPK), *dbl-1* and *sma-3* (TGF-β-like), and *daf-2* (insulin-like), according to real-time PCR [76].

Apart from these mechanisms, it is of interest how probiotics enhance the resistance to pathogens. One hypothesis is that the ability of probiotics to attach to the mucus layer and colonize in the *C. elegans* gut contributes to pathogen resistance. LGG is often used as a positive control in these studies because it has been reported to colonize well in the *C. elegans* gut and adhere to mucins (both in vitro and in vivo), which could constitute an important mechanism for probiotics. Indeed, increased colonization of *Lactobacillus sakei* 20D49 [77], *L. fermentum* JDFM216 [65, 78], *Lacticaseibacillus rhamnosus* GG [79], *Lacticaseibacillus rhamnosus* Probio-M9 [80], *Lactobacillus* spp. Lb21 [71], and multiple strains of *L. salivarius* and *L. plantarum* [41, 63] in the worm gut was associated with improved lifespan and/or resistance to enteropathogens like *E. faecalis*, *S. aureus*, *E. coli* O157:H7, *C. jejuni*, *S. typhimurium*, and MRSA. It is thought that probiotics outcompete pathogens and, thus, inhibit their colonization. For example, *L. acidophilus* NCFM [62], *L. salivarius* 13–7 [63], *L. salivarius* Z5 [63], *L. plantarum* (strains JDFM440, JDFM60, JDFM970, and JDFM1000) [41], *L. rhamnosus* GG [79], and *C. elegans*–derived *Stenotrophomonas* strain CPCC 101271 [81] inhibited the colonization of *E. faecalis, C. jejuni*, *S. typhimurium*/*E. faecalis, S. aureus*, and *Bacillus nematocida* B16, respectively, suggesting an inverse correlation between pathogen load and lifespan. However, in the case of CPCC 101271, although it improved lifespan by approximately 40% compared to OP50, *B. nematocida* outcompeted it and induced microbiota dysbiosis (according to metagenomic sequencing) [81]. Interestingly, despite *Enterococcus* being a source of infection in many models, multiple *E. faecalis* strains (12D26, 20D48, and 30D36) also have been suggested as potential probiotics as they enhanced resistance to *E. coli* O157:H7 and *S. aureus*, likely due to significant adhesion to mucin proteins and colonization in the *C. elegans* gut [77].

Conversely, other probiotics, such as *L. zeae* LB1 [82], *Lactobacillus* spp. Lb21 [71], or *Pediococcus* isolates (*P. acidilactici* DM-9, *L. brevis* SDL1411, and *P. pentosaceus* SDL1409) [83] did not significantly reduce colonization of ETEC, MRSA, or *P. aeruginosa* PA14, respectively, indicating that the mechanisms of these probiotics were not related to reduced pathogen load. Instead, they exerted anti-pathogenic effects through other mechanisms, including changes in gene expression and production of antimicrobial peptides (AMP) or other metabolites (i.e., nitric oxide [NO]), during infection. For example, *Pediococcus* species exhibited strong bactericidal properties (against *P. aeruginosa* PA14), which was likely due to the production of NO [83]. *C. elegans* are unable to intrinsically produce NO, but its production by probiotics may mediate immune responses [84]. In addition, *L. zeae* LB1 significantly suppressed the expression of ETEC toxin genes, including *estA*, *estB*, and *elt*, suggesting that decreased expression of these genes directly increased lifespan under ETEC infection [82]. Furthermore, *L. zeae* LB1 [67], *L. salivarius* 13–7 and Z5, and *L. plantarum* N9 [63] also increased the expressions of AMP genes including *lys-7* (lysozyme), *spp-1* (saposin-like protein), and *abf-3* (antibacterial factor) as well as upregulated defensin molecules like *abf-2* and *clec-85* (C-type lectin domain-containing protein). *L. plantarum* JBC5 also increased *lys-1*, *lys-8*, *spp-*7, *abf-2*, and *abf-3* [64]*. L. rhamnosus* GG enhanced the lifespan and pathogen resistance (*S. aureus*, *S. typhimurium*, and *E. faecalis*) and increased expressions of lysozymes and proteases involved in innate immunity [79]. Furthermore, gene ontology enrichment analysis confirmed the involvement of defense/innate responses (in particular to Gram-negative bacteria) in *L. rhamnosus* GG-mediated longevity [79]. Innate immune genes also require transcription factors to be activated—but these mechanisms have not been explored as much. Intestinal accumulation of HLH-26 after *E. faecium* treatment induced the transcription of immune genes including *ilys-2* (invertebrate-type lysozyme), *spp-2*, *cnc-2* (caenacin), and *clec-165* under *S. enterica* infection, suggesting that this transcription factor was responsible for the production of AMPs, which was associated with lifespan extension [85]. Collectively, these data suggest a positive correlation between transcript levels of AMP and defensin genes and *C. elegans* lifespan.

### Modulation of stress responses

Abiotic stressors like oxidation, heat, heavy metals, and UV irradiation can significantly damage lipids, proteins, and DNA, leading to dysfunction of cellular processes and, over time, a subsequent decline in health and QoL. Therefore, proper regulation of stress responses is critical and is another mechanism that probiotics may use to extend lifespan in *C. elegans*. Quenching of ROS by SOD and CAT is one component of the stress response in *C. elegans*. DAF (insulin-like) is a principal receptor and JNKs are a family of kinases and both have key roles in the *C. elegans* stress response and antioxidant pathways [35, 86]. Although the mechanism was not studied in vivo, *L. rhamnosus* R4 and *L. helveticus* S4 significantly improved lifespan compared to OP50 and *Enterococcus hirae* H4 and these lactobacilli demonstrated better scavenging activity of ROS and free radicals as well as improved cholesterol and triglyceride levels, in vitro, suggesting that antioxidant capacity may contribute to R4- and S4-mediated longevity [87]*.* Similarly, *L. plantarum* As21 feeding improved resistance to heat and hydrogen peroxide stresses, which was associated with decreased ROS levels and upregulation of SOD, CAT, and glutathione (GSH) [88]. *B. adolescentis* recapitulated the effects seen in *D. melanogaster* and significantly increased the lifespan of *C. elegans*, through *sod-3*- and *ctl-2*-dependent mechanisms [49]. Taken together, although signaling pathways were not heavily explored in these studies, scavenging of ROS or heat-induced stress molecules by antioxidants or enhanced resistance to exogenous stress may be critical features of probiotics and may improve lifespan via modulation of the stress response. However, it is important to consider that removal of ROS and reducing cellular stress by antioxidants as a mechanism of lifespan extension by potential probiotics may have no benefits or may even be detrimental [89]. For example, supplementation of GSH and its precursor *N*-acetyl cysteine accelerated aging and shortened lifespan in *C. elegans* by activating the unfolded protein response and increasing proteotoxic stress levels [90]. One speculation is that chronic or excessive supplementation of antioxidant compounds may interfere with normal, physiological ROS signaling, which, in turn, accelerates aging [90, 91]. Nonetheless, studying antioxidant capacities of probiotics and the effects of this mechanism on lifespan extension in *C. elegans* warrant further investigation.

In terms of mechanism, specific antioxidant target genes of *daf-16* include *sod-3* and multiple catalase (*ctl*) genes. Furthermore, *skn-1* is relevant in the stress response and is activated via phosphorylation downstream of *daf-2* by factors such as ROS and lipid accumulation [61]. Stress modulatory effects of *L. rhamnosus* CNCM I-3690 [92], *L. fermentum* U-21 [93], *L. fermentum* MBC2 [78], and *Lactobacillus gasseri* SBT2055 [94] in response to oxidative/heat stress were mediated, at least in part, by *daf*/*skn-1*, *jnk-1* signaling, or the *pept-1* gene (*L. fermentum* MBC2). Changes in these signaling pathways was also associated with higher mitochondrial numbers (*L. gasseri*), upregulation of *sod* and other stress regulatory genes, such as *hsp* (*L. rhamnosus* and *L. fermentum*), and changes to lipid metabolism (*L. rhamnosus* and *L. gasseri*). A screening of many potential probiotics revealed that *L. plantarum* 427, *Lactobacillus crispatus* X13, *Lactobacillus reuteri* 9–5, *L. fermentum* 422, *L. salivarius* Z5, and *L. reuteri* G14 significantly protected *C. elegans* from hydrogen peroxide- and juglone-induced oxidative stress, extending lifespan and upregulating activities of SOD, CAT, and malondialdehyde, decreasing ROS levels, and upregulating gene expressions of *pmk-1* and *sek-1* (MAPK signaling) as well as *skn-1* and *sod-3* (DAF signaling) [95]. Therefore, supplementation of these probiotics may have impacted stress response by enhancing resistance to exogenous stress and by reducing ROS or other markers of cellular stress. Multiple species of *Weisella*, including *W. koreensis* and *W. cibaria*, significantly extended lifespan, which was associated with decreases in ROS and ATP levels and significant upregulation of *daf-16*, *sod-3*, *jkk-1*, and *jnk-1* [96]. These data suggest the involvement of the JNK stress response as well as insulin-like signaling and, due to increased nuclear localization of *daf-16* (and decreased cytosolic concentration), the authors speculated that *Weisella* enhanced lifespan primarily through insulin-like signaling [96]. *daf* genes, including *daf-16, daf-12*, and *daf-7*, were significantly upregulated after treatment with *Streptococcus thermophilus* ST-T1 or ST-510 [97]. Hydrogen peroxide was also significantly lower in these worms, suggesting that its accumulation was suppressed by antioxidants, including SOD, CAT, or GSH. Indeed, according to real-time PCR, the expressions of *sod-3*, *sod-4*, *ctl-1*, and *ctl-2* were significantly elevated in *S. thermophilus*–treated worms and the authors concluded that the *daf-16* antioxidant pathway primarily contributed to lifespan extension [97]. Feeding of newly isolated and potential geroprotectors *Levilactobacillus brevis* and *Weizmannia coagulans* was associated with changes in gut barrier functions and increased resistance to exogenous stress (oxidative and heat), which was mediated, at least in part, by a combination of p38 MAPK, DAF (insulin-like), and JNK (more prominent in *W. coagulans*) signaling [98].

These studies highlight the importance of modulating stress responses to improve life- and healthspan in *C. elegans*. Activation of stress response pathways is critical during acute stress to prevent stress-induced damage to mitochondria, proteins, and DNA; this damage impairs normal cellular processes, including food intake behaviors and metabolism, which constitute another principal mechanism of probiotic-mediated geroprotection.

### Modulation of food intake and metabolism

It has been established that DR, or a reduction in food intake that does not result in malnutrition, is highly beneficial for extending lifespan and slowing aging progression in multiple models, including yeast, *D. melanogaster*, C*. elegans*, and rodents [99, 100]. In *C. elegans*, *eat-2* and *eat-18* mutant worms, which are defective in motor neurons related to pharyngeal pumping and, therefore, mimic DR during food intake, are well-established models for understanding how DR impacts metabolism [101]. Apart from *eat* genes, other central regulators of energy balance and nutrient sensing in *C. elegans* include the *daf* (insulin-like) pathway, adenosine monophosphate-activated kinase (*aak-2*), NAD^+^-dependent deacetylases (i.e., *sirt-1*), and *skn-1* and *pha-4* (defective in pharynx development) transcription factors [102–105]. Feeding of *P. acidilactici* MNL5 improved lifespan through a DR-mediated mechanism as the worms exhibited a significantly smaller body size compared to their OP50-fed counterparts [106]. The authors hypothesized that bile salt hydrolase (BSH) activity of *P. acidilactici* may have driven its benefits, which also were associated with decreased accumulation of lipid droplets and suppression of *fat-4*, *fat-5*, and *fat-6* genes, which are related to fat storage in *C. elegans*. Furthermore, *Bacillus subtilis* spores and vegetative cells (strains PXN21, JH642, 168, and NCIB3610) protected against α-synuclein aggregation (hallmark pathology of Parkinson’s disease) through DR-dependent mechanisms [107]. In particular, *eat-2* mutants harbored significantly less aggregates per worm when given OP50 or *B. subtilis* and *daf-16* mutation led to a rapid increase in aggregates when given vegetative *B. subtilis.* Spores attenuated neurotoxic protein aggregation through a *pha-4*-dependent DR mechanism. However, more studies should be conducted to better understand the differing effects between vegetative and sporulated *B. subtilis*.

Feeding behaviors directly regulate metabolic pathways, including those associated with the breakdown of fatty acids/lipids, proteins, and carbohydrates. Metabolism of these biomolecules facilitates cellular respiration through the tricarboxylic acid (or TCA) cycle and ion exchange, which ultimately regulate energy intake and expenditure [108]. High-throughput “omics” analyses, including metabolomics and proteomics, coupled with in vivo screening approaches, allow correlation between physiological changes and molecular changes/pathway analysis, which improves our understanding of how certain bacteria are impacting lifespan and through what mechanisms. Untargeted nuclear magnetic resonance-based metabolomics revealed that geroprotective effects of *Lactobacillus* spp. Lb21 were associated with increases or changes in metabolites related to energy metabolism and oxidative stress/osmoregulation, suggesting the importance of metabolic flux in regulating lifespan and health [109]. Furthermore, high accumulation of *Lactobacillus delbrueckii* subsp. *bulgaricus* in the *C. elegans* gut (compared to two *L. delbrueckii* subsp. *lactis* isolates and OP50) was associated with lifespan extension as well as increased synthesis/accumulation of folate and amino acids and secretion of galactose (indicating upregulated galactose metabolism) and decreased saturated fatty acids [110]. Inhibition of folate synthesis by bacteria or its activity in *E. coli*–fed worms has been shown to accelerate aging and worsen longevity, suggesting the importance of folate (in the presence of certain bacteria) for mediating life- and healthspan [111, 112]. Furthermore, decreases in saturated fatty acids, such as palmitic acid and stearic acid, as well as enhanced galactose metabolism, have been associated with increased lifespan in other studies and likely influenced longevity mediated by *L. delbrueckii* subsp. *bulgaricus* here [110, 113, 114]. Overall, bacterial metabolism of lipids, proteins, and carbohydrates, is a fundamental mechanism contributing to lifespan extension and geroprotection. However, these mechanisms, especially in the context of potential probiotics, remain largely unexplored and require further investigations.

### Lifespan extension via multiple mechanisms

As with any biological system, *C. elegans* are complex and, as such, many mechanisms contribute to probiotic-mediated lifespan extension and geroprotection. In most cases, innate immunity, stress responses, and energy metabolism/DR (among others) work together to impact host physiology, but few studies have discussed this interplay in *C. elegans*, particularly in the context of probiotic-mediated effects. There are connections between the oxidative stress response and lipid metabolism and *B. animalis* subsp. *lactis* CECT 8145 feeding modulated both of them [115]. This probiotic enhanced resistance to oxidative stress but also significantly upregulated energy metabolism (correlated with increases in carbohydrate, lipid, and xenobiotic/drug metabolisms) through *daf-2* and *daf-16* signaling [115]. Feeding of *P. acidilactici* CECT9879 also modulated *daf* genes, downregulating *daf-2* and upregulating *daf-16* [116]. Upregulation of *daf-16* and its nuclear localization was associated with reduced ROS accumulation, increases in genes related to fatty acid degradation (i.e., β-oxidation genes in mitochondria and peroxisomes), and a suppression of genes responsible for fatty acid synthesis (i.e., *fasn*, *fat-5*, *fat-7*, and *mdt-15*) [116]. Similarly, extension of lifespan in *L. salivarius* FDB89-fed worms was correlated with higher SOD activity and XTT reduction (related to metabolism), which naturally decline with age [117]. *L. salivarius* feeding was also associated with smaller body size, lower reproductivity, and a significant decline in pharyngeal pumping rate, suggesting that both stress response and DR mechanisms contributed to *L. salivarius–*mediated longevity [117].

On the other hand, centenarian-derived *B. longum* BB68 impacted both innate immunity and oxidative stress mechanisms to extend lifespan by 28% and this was dependent on *daf-16* mediated transcription of antioxidant and anti-aging genes [118]. Lifespan promotion was also dependent on *tir-1* and *jnk-1*, suggesting that TIR-JNK signaling, and their crosstalk are central to BB68-mediated longevity via *daf-16.* Similarly, *W. confusa* demonstrated geroprotective effects in comparison to OP50 and also under hydrogen peroxide-induced oxidative stress and *S. typhimurium* infection [119]. Mechanistically, *W. confusa* had significantly greater free radical and superoxide scavenging capabilities and RNAseq confirmed that glutathione S-transferase genes (*gst-44*, *gst-9*, and *gst-18*, involved in detoxification) and *sod-5* were significantly upregulated and differentially expressed [119]. Feeding with *Lactococcus cremoris* subsp. *cremoris* also mediated significant protection from heat- and juglone-induced cellular stresses through *skn-1* [120]. Notably, under *S. enteriditis* and *S. aureus* infections, *L. cremoris* feeding significantly extended lifespan, which was lost in *daf-16*, *skn-1*, and *pmk-1* mutants, suggesting involvement of innate immunity, stress responses, and potentially metabolic flux in *L. cremoris*–mediated longevity [120].

Furthermore, lifespan improvement by *L. rhamnosus* Probio-M9 (isolated from healthy breast milk) was associated with modulation of innate immune signaling, stress responses, as well as metabolism [80]. For example, enhanced resistance to heat stress was likely dependent on *skn-1* but Probio-M9 lifespan extension also was at least partially dependent on p38 MAPK genes (*nsy-1*, *sek-1*, and *pmk-1*) and associated with an increased mitochondrial unfolded protein response as well as the metabolism of amino acids, sphingolipids, fatty acids, and galactose [80]. Genes related to lipid metabolism (*fat-4* and *lipl-4*) and antioxidants (*sod-3*) were also upregulated in worms fed *P. acidilactici* and the authors speculated that *P. acidilactici* was immunoregulatory due to antimicrobial effects and production of bacteriocin [121]. Furthermore, lifespan extension was lost in *daf-2*, *daf-16*, and *jnk-1* mutants, suggesting that *P. acidilactici*–mediated effects were at least partially dependent on insulin-like and JNK signaling. Lastly, feeding of *C. butyricum* MIYAIRI 588 (a potent producer of butyrate, a well-studied SCFA) alone and in combination with OP50 enhanced resistance to *S. enterica* and *S. aureus* pathogens as well as UV irradiation-mediated cellular stress, and reduced body size, suggesting modulation of innate immunity, stress response, and DR by *C. elegans* [69]. In fact, these effects were at least partially dependent on *daf-*2, *daf-16*, and the *skn-1*/*nrf-2* pathway. Taken together, these data suggest that *L. rhamnosus* Probio-M9–, *P. acidilactici*–, and *C. butyricum*–mediated lifespan extension may be dependent on modulation of multiple *C. elegans* pathways.

### Other mechanisms

Some bacteria also utilize other mechanisms apart from the immune system, stress response, or food intake and metabolism. For example, four strains of *Bacillus licheniformis* (141, 143, 147, and 156) significantly increased lifespan compared to OP50 and *B. subtilis* control strains, and it was revealed that genes involved in serotonin signaling (i.e., *tph-1*, *bas-1*, *mod-1*, and *ser-1*) were upregulated [122]. This is of interest because it is known that serotonin signals the presence of food and, therefore, modulation of this signaling pathway may extend lifespan and be geroprotective by mimicking DR [123]. However, more studies need to be conducted to better understand the geroprotective effects of probiotics in the context of serotonin signaling.

One notable property of *B. subtilis* strains, including JH642, NCIB3610, RG4365, PXN21, and 168, is that they are spore-forming bacteria, meaning that they are able to remain dormant as spores in the gut and in the absence of adequate nutrients [124]. In wild-type and transgenic worms of neurodegenerative disease (i.e., Parkinson’s and Alzheimer’s diseases), *B subtilis* strains in both the vegetative (active) and spore (dormant) stages remained colonized in the gut and improved lifespan, through *daf-16* (insulin-like) and DR [107, 124, 125]. The formation of biofilm and production of NO by these *B. subtilis* strains is another prominent mechanism that was explored in these studies. Specifically, *B. subtilis* defective in *tasA*, *espG*, and *bslA* (biofilm formation-related genes), as well as the *nos* mutant (defective in NO production) were unable to extend lifespan, suggesting the longevity effects were associated with biofilm formation and/or NO production [124]. The formation of biofilm and production of NO protected from α-synuclein accumulation (a major pathology observed in Parkinson’s disease) [107] and delayed neuronal deterioration, as well as ameliorated from paralysis and poor chemotaxis in worms overexpressing amyloid-β (a major pathology of Alzheimer’s disease) [125].

Another interesting, yet largely unexplored, mechanism of probiotics in the context of lifespan extension is improvement in gut barrier functions. It has been found that many probiotics confer pathogen or stress resistance or modulate metabolic functions by significant adhesion to mucins and colonization in the gut. In fact, a major criterion to be considered a probiotic is the ability to survive in acidic/gastrointestinal conditions [126]. However, a less studied phenomenon is the ability of these bacteria to modulate intestinal permeability, through regulation of tight junction proteins or mucins. As we age, there is a natural deterioration in gut barrier integrity (called “leaky gut”), which is associated with gut dysbiosis, breakdown of tight junction proteins, and a thinning of the mucin layer [127]. Exposure to infectious agents, increased cellular stress, and irregular metabolism may accelerate this aging-related phenotype and, thereby, also elevate gut permeability and impair normal gut functions. Therefore, modulation of the gut microbiota by probiotics may restore gut barrier functions and decrease gut permeability and we have shown this in mammalian cell culture systems, *C. elegans*, and mice [27]. Specifically, although we did not focus on lifespan extension in that study, we found that probiotics may have exerted beneficial effects on intestinal permeability via BSH, a bacteria-derived enzyme that deconjugates bile salts [27]. In the context of *C. elegans* lifespan, *P. acidilactici* MNL5 may have extended lifespan and strengthened gut barriers through similar BSH activity, which subsequently modulated lipid metabolism and DR [106]. Also similar to our reports, *L. cremoris* subsp. *cremoris* [120], *Levilactobacillus brevis* [98], and *W. coagulans* [98] all attenuated leaky gut and improved gut barrier integrity, which was measured by quantifying the leakage of blue dye from the gut cavity (Smurf assay) or using transgenic (*dlg-1*::GFP-labeled) worms.

Here, we have reviewed recent research reporting the anti-aging and potential geroprotective effects of probiotics and how modulation of principal signaling pathways, including innate immunity, stress responses, and metabolism, is central to probiotic-mediated effects. Most studies identify *Lactobacillus* and *Bifidobacterium*, but emerging studies are identifying new probiotic strains. Despite the literature reviewed here and the promising findings reported, there is still a lack of high-throughput screenings of potential probiotics, which will better elucidate strain-specific effects and add to our understanding of their geroprotective potential.

## Geroprotective effects of postbiotics in *C. elegans*

Most of the research to date has focused on the effects of probiotics, or live bacteria. However, studies involving postbiotics and their classification are emerging, especially in the contexts of obesity/diabetes and aging [32]. Postbiotics are defined as “dead” or “heat-inactivated/pasteurized” probiotics and their byproducts/metabolites, which confer health benefits on the host [12]. Although not studied as extensively as probiotics, there is emerging research describing the geroprotective potential of postbiotics in *C. elegans*. One of the first postbiotic studies screened approximately 350 strains of heat-killed lactic acid bacteria, which had been isolated from stool samples and kimchi, and *L. fermentum* LA12 and *L. plantarum* CJLP133 were found to significantly improve lifespan compared to heat-killed OP50 and LGG—however, the mechanisms were not investigated [128]. Later, these researchers reported that heat-killed *L. plantarum* 133 and *L. fermentum* 21 significantly improved lifespan in *S. typhimurium*– and *Yersinia enterocolitica*–infected worms, which was also associated with upregulation of innate immunity genes involved in *pmk-1* signaling (i.e., *acdh-1* [acyl-CoA dehydrogenase] and *cnc-2*) and some downstream effectors of *daf-16* (i.e., *dod-19*) [129]. Similarly, heat-inactivated *Lactobacillus curvatus* BGMK2-41 feeding demonstrated immunoregulatory effects as it enhanced pathogen resistance against *S. aureus* ATCC 25923 and *P. aeruginosa* PA14. Pathogen resistance and lifespan extension was correlated with upregulation of *tir-1*, *pmk-1*, and *atf-7* and antimicrobial genes (AMPs, C-type lectins, and lysozymes), according to real-time PCR, and increased phosphorylated p38 MAPK (according to Western blots), suggesting that BGMK2-41 effects were dependent on modulation of this pathway [130]. Our laboratory reported that dead *L. paracasei* D3.5 significantly promoted *C. elegans* lifespan and aging-related parameters (i.e., muscle mass and movement decline) via cell wall–derived lipoteichoic acid [32]. We did not investigate the *L. paracasei*–mediated mechanisms in worms, but we reported that TLR-2/p38 MAPK signaling was activated by the postbiotic in older mice, indicating that anti-inflammatory and innate immune response modulation may be principal mechanisms contributing to lifespan extension by D3.5 [32].

Regarding modulation of stress response, heat-killed *B. longum* significantly increased survival during heat- and hydrogen peroxide-induced oxidative stress [131]. Fluorescent microscopy revealed nuclear localization of *daf-16* and real-time PCR demonstrated upregulation of *daf-16* mRNA as well as other stress response and antioxidant genes, including *hsp-12.6*, *hsp-70*, *skn-1*, *ctl-1*, *ctl-2*, and *sod-1* [131]. Mitochondrial function, as assessed by increases in mitochondrial ROS levels, membrane potential, and activity, was important for extending lifespan by *B. longum* and *L. fermentum* BGHV110 postbiotics [131, 132]. The effects of heat-killed *L. fermentum* were driven by HLH-30, which is a transcription factor regulating expressions of autophagy-related genes, such as lysosomal hydrolases and membrane proteins [132]. Therefore, increased autophagy of aging or senescent cells may have improved lifespan in *L. fermentum* postbiotic-fed *C. elegans*, as this has been studied as a mediator of lifespan extension in multiple studies and organisms [133]. Recently, cell-free supernatants of *Akkermansia muciniphila* extended lifespan while reducing ROS levels and oxidative damage [134]. *A. muciniphila* postbiotic also modulated metabolic functions, which was indicated by upregulation of glucose metabolism (i.e., *gsy-1*, *pygl-1*, and *pyk-1*) and lipid metabolism (i.e., *acs-2*, *cpt-4*, and *tph-1*) genes and, conversely, a downregulation of *fat-7*, which regulates fatty acid biosynthesis [134].

Furthermore, some of the probiotics reported above also extended lifespan and demonstrated geroprotective potential when fed to *C. elegans* in the heat-/UV-killed form. For example, *L. cremoris* subsp. *cremoris* [120], *B. infantis* [74], *B. animalis* subsp. *lactis* CECT 8145 [115], *C. butyricum* MIYAIRI 588 [69], and four isolates of *B. subtilis* (strains PXN21, JH642, 168, and NCIB3610) [124] improved lifespan when administered in both the live and non-viable forms, suggesting that cellular components or bacterial metabolites (such as SCFAs or bile acids), mediate probiotic and postbiotic effects in *C. elegans*.

Despite these exciting findings, there is still a large gap in our understanding of postbiotics and their effects on aging, both physiologically and mechanistically. It is well studied that probiotic bacteria and their ingredients (i.e., cell wall components or cell surface molecules) and/or the antimicrobial molecules they secrete elicit innate immune or stress responses, which contributes to enhanced resistance against pathogens and heat/oxidative stress and improved lifespan; perhaps these are the major components driving postbiotic-mediated geroprotection [132]. Furthermore, since some studies have reported that postbiotic effects are not mediated by increased colonization in the *C. elegans* gut [130], the beneficial effects could be related to upregulation of gut barrier proteins (i.e., tight junction proteins) [135]. Changes in gut barrier functions have been implicated as a potential mechanism of both probiotics and postbiotics, but still requires investigation. It is also important to conduct additional studies because it is still unclear why some postbiotics have demonstrated geroprotective effects, but others are only beneficial when administered in the live form, such as with *L. rhamnosus* Probio-M9 and *E. faecium* [80, 85].

## Future perspectives

This article has reviewed the geroprotective potential of probiotics and postbiotics in *C. elegans* and their major mechanisms. *C. elegans* are a valuable model for high-throughput drug and probiotic/postbiotic screens; however, they lack several organ/systems present in mammals, such as a brain and vasculature, which severely limits aging studies [136]. Therefore, in order to apply findings from *C. elegans* screenings to human health, it is important to validate their findings in mammalian systems, like mice, to understand the anti-aging and geroprotective potential of microbiome modulators on human biology. It is also important to use complex systems that harbor a gut microbiome so we can gain a more complete understanding of probiotic- and/or postbiotic-mediated gut microbiota modulation and how these shifts impact lifespan extension and geroprotective pathways (innate immunity, stress response, and metabolism).

Future studies should also investigate the anti-aging potential of multi-strain probiotics, which may exert synergistic effects when administered in combination. Multi-strain probiotics have been largely studied using models of cancer, enteric infection, and obesity/diabetes [20, 137–142], but the effects of multi-strain probiotics on aging remain elusive. Some emerging studies are reporting that other nutrients and dietary supplements, like folic acid, vitamin D, vitamin B_12_, and cranberries improve longevity in *C. elegans* due to their antioxidant effects [143–146]. Based on these interesting findings, future studies should continue to investigate the geroprotective potential of other dietary supplements, such as prebiotics/resistant starches (indigestible fibers that serve as food for probiotics [i.e., inulin and sago]), synbiotics (the synergistic formulation of probiotics and prebiotics), and fermented foods (i.e., yogurt). Previous studies have reported the antidiabetic effects of these supplements in preclinical models and clinical trials, but their anti-aging and geroprotective potentials remain unknown [21, 147, 148]. Elucidating the geroprotective potential of food supplements is also of interest because many of the probiotics/postbiotics reviewed here were isolated from fermented foods like dairy and kimchi and fermented foods may be a better approach for integrating probiotics or bioactive ingredients into the diet to extend life and improve health and QoL. Therefore, screening them for their anti-aging and geroprotective effects using *C. elegans* will serve as a gateway to future preclinical and clinical studies, which will greatly contribute to the development of therapeutics and food supplements that will increase lifespan, slow aging progression, and improve QoL.

## Conclusions

Microbiome modulators, such as probiotics and postbiotics, have strain-specific geroprotective effects in *C. elegans*. Live and non-viable (pasteurized) bacteria from many genera including *Lactobacillus*, *Bifidobacterium*, *Enterococcus*, *Bacillus*, and others promote longevity and have geroprotective potential via modulation of multiple pathways, such as (1) innate immunity, (2) stress response, and (3) DR and regulating metabolism (i.e., of fatty acids/lipids, amino acids, and carbohydrates). Despite the findings reviewed here, additional high-throughput screening studies are needed to elucidate the interplay of these multiple mechanisms and understand the anti-aging and geroprotective potentials of probiotics and postbiotics. Furthermore, screening multi-strain probiotics, prebiotics/resistant starches, synbiotics, and fermented foods will add to our understanding of the potential benefits and anti-aging effects of these supplements and potential geroprotectors, which can be a promising therapeutic strategy for improving lifespan and reducing the risk of aging-related diseases and their burden.

## Abbreviations

AMP: Antimicrobial peptide
BSH: Bile salt hydrolase
CAT: Catalase
*C. elegans*: *Caenorhabditis elegans*
*D. melanogaster*: *Drosophila melanogaster*
DAF: Dauer formation
DR: Dietary restriction
ETEC: Enterotoxigenic *Escherichia coli*
GSH: Glutathione
JNK: C-Jun N-terminal kinase
LGG: *Lactobacillus rhamnosus* GG
MAPK: Mitogen-activated protein kinase
MRSA: Methicillin-resistant *Staphylococcus aureus*
NO: Nitric oxide
OP50: *Escherichia coli* OP50
QoL: Quality of life
ROS: Reactive oxygen species
SCFA: Short-chain fatty acid
SOD: Superoxide dismutase
TGF-β: Transforming growth factor-β
TLR: Toll-like receptor
